# From safety net to trampoline: elevating learning with growth mindset in healthcare simulation

**DOI:** 10.1186/s41077-023-00264-1

**Published:** 2023-11-10

**Authors:** Samantha Rae Hopkins, Valerie Isobel Rae, Samantha E. Smith, Stephen Meldrum, Victoria R. Tallentire

**Affiliations:** 1 Medical Education Directorate, NHS Lothian, Edinburgh, UK; 2 Scottish Centre for Simulation and Clinical Human Factors, NHS Forth Valley, Larbert, UK; 3 University of Edinburgh, Edinburgh, UK

**Keywords:** Growth mindset, Psychological safety, Simulation

## Abstract

The *Implicit Theory of Mindset* proposes two different mindsets that sit at opposite ends of a spectrum: a fixed mindset versus a growth mindset. With a fixed mindset, an individual believes they are born with a certain amount of an attribute, and so their potential is both pre-determined and static. With a growth mindset, an individual believes their attributes are malleable and can strengthen over time with repeated effort, adaptable learning strategies, and challenge seeking. Adoption of a growth mindset is associated with improved academic success, more effective learning strategies, increased resilience in the face of adversity, and better mental wellbeing.

The theoretical underpinning of psychological safety resonates with the *Implicit Theory of Mindset* as it infers that a significant number of simulation participants have a fixed mindset and are therefore more likely to be fearful of making an error. The simulation community agree that participants need to feel comfortable making errors for simulation to be successful. The key word here is *comfortable*. Participants feeling *comfortable* to make errors just scratches the surface of adopting a growth mindset. With a growth mindset, participants see errors as a *positive* in the simulation experience, an inevitability of the learning process, evidence that they are adequately challenging themselves to improve.

Encouraging adoption of a growth mindset in participants is a powerful addition to the establishment of psychological safety because a growth mindset will re-frame participants’ experiences of social comparison from negative to positive and optimize information processing. We propose a novel idea: simulation educators should be explicit in the pre-brief about what a growth mindset is and its associated benefits to encourage its adoption during the simulation activity—a simulation growth mindset intervention. If this is not possible due to time constraints, an online module or article about growth mindset would be appropriate as pre-reading to encourage adoption of a growth mindset in participants. The message is not that a simulation growth mindset intervention should replace the focus on psychological safety but rather that it should be used synergistically to provide the highest quality simulation experience.

## Background

### Introduction

Johnny enters the simulation suite. The facilitator is warm and gracious; she makes everyone in the room feel safe from judgment and reproach. Johnny understands that if he makes an error, no one is going to make him feel incompetent in his professional role. The problem, however, is that Johnny has a persistent inner monologue that leaks like a faulty tap in his head. His inner monologue reminds him to focus on his performance so that he receives external validation from the facilitator and participants, which reinforces that he is intelligent and talented. The inner monologue persuades him to volunteer for scenarios where he already feels intelligent and accomplished, to reduce the chances of him making an error and reminding himself of his inescapable deficiencies. The inner monologue encourages him to feel shame when other participants perform better than him. The problem for Johnny is that although he feels the environment is psychologically safe, he has an inner belief system that limits his ability to extract the full learning potential from the simulation activity.

Psychological safety is a cornerstone of simulation-based education (SBE). The rationale is that it creates an environment that is conducive to interpersonal risk taking [1, 2] and reduces the emotional threat of tarnishing participants’ professional identity if they make an error [3–5]. Psychological safety also focuses on building rapport to ensure participants’ expectations, goals, and feelings are valued and respected. If psychological safety and rapport are neglected, participants are more likely to feel shame, anger, and disengagement from the simulation activity [6]. In this debate article, we will argue that the goals and benefits of psychological safety can be amplified by the addition of a growth mindset intervention.

### Growth mindset

In 1988, American psychologist Carol Dweck introduced her *Implicit Theory of Mindset*, and it spread like wildfire in the world of education [7]. A mindset is a belief one has about their attributes, such as intelligence or procedural ability. The theory proposes two different mindsets that sit at opposite ends of a spectrum: a fixed mindset versus a growth mindset. With a fixed mindset, an individual believes they are born with a certain amount of an attribute, and so their potential is both pre-determined and static. The focus is on performance-orientated goals, striving to surpass others (performance-approach) or avoiding performing worse than others (performance-avoidance) [8]. With a fixed mindset, you are also more likely to be afraid of making an error as it reveals your permanent deficiencies. With a growth mindset, an individual believes their attributes are malleable and can strengthen over time with intentional and repeated effort, adaptable learning strategies and challenge-seeking. With a growth mindset, you think your potential is infinite because your attributes have limitless growth [9]. The focus is on learning-orientated goals, prioritizing the significance of acquiring new knowledge and skills, rather than measuring yourself against others [8].

The *Implicit Theory of Mindset* is relevant to the simulation community because some simulation participants may have a fixed mindset [10–13]. Participants who adopt a fixed mindset participants may be more fearful of making mistakes, receiving negative feedback, and having an inferior performance to peers, because these highlight their perceived innate and permanent deficiencies. The establishment and maintenance of psychological safety aims to mitigate these negative outcomes, by making participants feel comfortable to make errors and create an environment in which reflection and feedback from peers are given with consideration for the recipients’ emotional wellbeing [2, 6]. Encouraging adoption of a growth mindset in participants has more ambitious goals with regards to the role of both errors and feedback.

### Literature overview

There has been a vast amount of research identifying the benefits to learners across disciplines and ages when adopting a growth mindset. Most of these studies aimed to establish causation through randomization of learners to a growth mindset intervention versus a control group and evaluating outcomes. A growth mindset intervention is an umbrella term for providing information to learners that attributes can grow when an individual exerts effort toward a challenge. Often this information includes a summary of research on brain neuroplasticity and the benefits of adopting a growth mindset [14]. When learners receive a growth mindset intervention, they are more likely to have improved academic success [15], better learning strategies [16], increased resilience, and enhanced mental wellbeing outcomes [10–13, 17].

Although growth mindset adoption is positively correlated with academic achivement in 72 out of 74 developed countries, there is no positive correlation in China and Lebanon [18]. Yeager and Dweck hypothesize that the lack of positive correlation in China could be because a growth mindset cannot further increase study hours or test scores when there is already a strong cultural expectation to work hard [19]. However, this does not indicate that a growth mindset has no effect on mental wellbeing in China. Students in China exhibit a pronounced connection between a fixed mindset and the “fear of failure” [18], which is a precursor to negative mental wellbeing outcomes [10–13]. In summary, although growth mindset adoption may have differing benefits in different cultural contexts, it is still likely to benefit learners across the globe.

Most of the research focusing on the *Implicit Theory of Mindset* in healthcare education are correlation studies rather than causation studies. In these studies, researchers evaluate participants’ pre-existing mindset using a validated Growth Mindset Scale [9] and correlate with other variables, such as mental wellbeing, effective use of learning strategies, self-reported medical errors, and academic achievement [9]. A fixed mindset in veterinary and medical students has been associated with increased stress and anxiety, and a growth mindset in this same group was associated with improved psychological wellbeing [10–12]. In nursing students, adoption of a growth mindset is correlated with increased usage of evidence-based learning strategies [16, 20].

In 2016, it was estimated that medical errors rank as the third leading cause of death in the USA [21]. Interestingly, studies in healthcare professionals around this period show minimal or no correlation between growth mindset and self-reported medical errors [22]. The self-reporting of medical errors may have been underestimated due to fear of social stigma.

The literature is sparse on the impact of participant mindset in the context of SBE, but there are some promising studies demonstrating positive causations after growth mindset interventions. One study demonstrated that healthcare professionals receiving a growth mindset intervention prior to a computer game training simulation (neonatal resuscitation) had improvement in longitudinal performance compared to a control group [23]. Two randomized studies demonstrated that instructional messages—designed to influence mindset—can improve procedural performance and motivation for secondary school students performing simulation-based procedural skills [24]. At the University of Melbourne, Jill Klein and her team flipped the script. Instead of using growth mindset interventions with the aim of improving participant performance or motivation in the simulation, they used the simulation itself to teach growth mindset as a coping strategy for medical students in the context of medical error [25].

## Novel idea

What if we could empower our simulation participants to adopt a growth mindset rather than focusing on mitigating the negative outcomes of a fixed mindset? We propose that simulation educators be explicit in the pre-brief about what a growth mindset is, and its associated benefits, to encourage adoption of a growth mindset during the simulation activity—a simulation growth mindset intervention. A growth mindset intervention could be as compact as a 10-min presentation, introducing participants to the concept of a growth mindset and why they might find it helpful. If this is not possible during the pre-brief due to the time constraints on the session, pre-reading would be appropriate. While details of growth mindset interventions are outside the scope of this article, further information about specific interventions can be found in a recent review by Burnette et al. [14].

The aim of the simulation growth mindset intervention is to encourage adoption of a growth mindset in participants and thereby maximize the success of the simulation activity. The establishment of psychological safety provides a safety net for simulation participants; we believe a growth mindset intervention will be a trampoline to elevate learning, performance, motivation and mental wellbeing.

## Benefits

We will discuss the benefits of a simulation growth mindset intervention in re-framing participants’ experience of social comparison and to optimize participant information processing.

### Social comparison

The establishment of psychological safety aims to make a simulation participant feel comfortable to perform when being observed. It does not consider the impact on the individual when observing other participants’ performances. When participants do not have a growth mindset, they can see other participants’ success in simulation as a threat if they do not perform as well or better because it reveals, by comparison, their own innate and permanent deficiencies [7]. We believe this feeling of helpless inferiority can be damaging to engagement in the simulation activity, and the research supports that this can be damaging to a participants’ mental wellbeing [10–13, 26]. If we do not make participants aware of the benefits of adopting a growth mindset, the simulation experience could contribute to psychological damage with negative consequences such as avoidance of further simulation activities and burnout.

In contrast, if a participant is empowered to adopt a growth mindset during the simulation activity, they may feel less threatened if other participants have a superior performance. In fact, if they adopt a true growth mindset, they should perceive other participants’ success as inspiration for what they can achieve with dedication, effort, and effective learning strategies [9]. Inclusion of a simulation growth mindset intervention is essential for the simulation to be successful because it shifts the needle of social comparison from a negative to a positive.

### Information processing

The purpose of many simulation activities is for participants to improve performance by learning new things. This is often achieved through feedback from facilitators and peers. Having a growth mindset enlarges the area of the brain associated with goal formation and decision-making and primes participants’ brains to process feedback [27, 28]. These processes are essential to enhance participant experience in simulation.

Using functional magnetic resonance imaging studies, neuroscientists have shown that people with higher growth mindset scores have differences in brain structure. Participants with higher growth mindset scores have increased grey matter volume in the medial orbitofrontal cortex, the part of the brain responsible for goal formation and decision-making [27]. Neuroscientists have also demonstrated that participants with growth mindsets have more brain activity in areas that process corrective information after an error [28]. When participants with a growth mindset give an incorrect answer to a question and receive feedback, their attention is fully focused to “regulate sensory and response selection” [28]—in other words, they are interested in finding out what the correct answer is and why. In contrast, participants with a fixed mindset who give an incorrect answer and receive feedback demonstrate higher levels of activity in the limbic system. The limbic system regulates emotional response to sensory information such as feelings of shame and despair [29]. In summary, when receiving feedback after an error, growth mindset participants have deep processing of new corrective information, whereas fixed mindset participants have a more emotional response. As participants will receive feedback from other participants and the facilitator(s) during SBE, how this information is received, processed, and acted upon is essential to optimize learning.

An individual’s mindset sits on a spectrum from fixed to growth and, even for one learner, is dependent on a particular set of circumstances. The establishment of psychological safety in SBE aims to make participants feel comfortable to make errors. Feeling comfortable to make errors is a halfway house between a growth and fixed mindset. With a growth mindset, you are not just comfortable making errors, you see errors as a positive in the learning process. This positive association with errors optimizes the brain processing of corrective information. If we want participants to squeeze all the potential learning out of the fruitful educational experience, we need to do everything in our power to prime participants’ brains for deep processing of feedback and enhanced goal setting and decision-making.

## Anticipated criticism

We will pre-emptively dispute the possible criticisms that a growth mindset intervention is patronizing for adult learners (undergraduate students or professionals) and may be poorly received by learners from non-Westernized backgrounds.

### Informative, not patronizing

Being explicit about the importance of a growth mindset could be perceived as patronizing to adult learners. Many educators will argue that adult learners have agency and should not be actively corralled into changing their beliefs or attitudes for the purpose of a simulation activity. Educators may argue that adult learners do not need simulation facilitators to push their educational agendas on to them.

We would first contend that there is a knowledge gap, and so participants are unable to appreciate the benefits of adopting a growth mindset for the simulation activity to be successful. Many healthcare professionals have never heard of a growth mindset because most education on growth mindset provided to learners is in the primary and secondary school setting in the Euro-American world [15, 30]. The *Implicit Theory of Mindset* does not appear to have permeated to healthcare professionals on the shop floor. This is not reiterating already-known information to participants but rather highlighting an important tool in their arsenal for both the simulation activity and their clinical practice.

As an adult learner, we want the facilitator to be upfront with their intentions. An idea that resonates with us is *above the table negotiation*, a communication technique that encourages educators to be explicit about what they want instead of expecting learners to have telepathic powers [31]. If facilitators are open and provide a simulation growth mindset intervention, they are empowering participants to make their own decision about adopting a growth mindset to enhance the quality of their simulation experience. Being honest with our participants is treating them like adults.

## Directions for future research

Although the literature on growth mindset interventions being incorporated into simulation-based education is sparse, there is ample evidence supporting the benefits of growth mindset interventions in educational settings. We therefore believe the focus of further research should be on the *type* of growth mindset intervention that will be most efficiently and effectively incorporated into SBE. This is unlikely to be a one-size-fits-all solution but rather an exploration of different methodologies, such as didactic teaching, educational reading materials, and/or written exercises, that simulation educators can incorporate into their simulation-activities [14]. The literature also supports that learners gain more benefit from a growth mindset intervention if educators themselves have a growth mindset [32, 33]. We believe further research is required to determine what is the most efficient and effective educator-based growth mindset interventions for simulation faculty development.

## Conclusion

Encouraging adoption of a growth mindset is notably absent from the conversation about psychological safety. We need to go further than participants feeling comfortable to make errors. We must empower them to see errors as a positive in SBE, an inevitability of the learning process. This can only be achieved by being explicit with our participants about what a growth mindset is and the benefits of adopting one. Let us put our cards on the table.

The final message is not that a simulation growth mindset intervention should replace the establishment of psychological safety in simulation but rather that it should be used synergistically. Let us use explicit strategies to encourage our participants to feel psychologically safe and adopt a growth mindset to enhance the quality of their simulation experience.

## Data Availability

Not applicable.

## Abbreviation

SBE: Simulation-based education

## References

[1] Edmondson A (1999). Psychological safety and learning behavior in work teams. Admin Sci Quart.

[2] Rudolph JW, Raemer DB, Simon R (2014). Establishing a safe container for learning in simulation: the role of the presimulation briefing. Simul Healthc.

[3] Ibarra H (1999). Provisional selves: experimenting with image and identity in professional adaptation. Admin Sci Quart.

[4] Pratt MG, Rockmann KW, Kaufmann JB (2006). Constructing professional identity: the role of work and identity learning cycles in the customization of identity among medical residents. Acad Manage J.

[5] Gilmore S, Anderson V (2012). Anxiety and experience-based learning in a professional standards context. Manage Learn.

[6] Auerbach M, Cheng A, Rudolph JW (2018). Rapport management: opening the door for effective debriefing. Simul Healthc.

[7] Dweck CS, Leggett EL (1988). A social cognitive approach to motivation and personality. Psychol Rev.

[8] Wolcott MD, McLaughlin JE, Hann A, Miklavec A, Dallaghan GBL, Rhoney DH (2021). A review to characterise and map the growth mindset theory in health professions education. Med Educ.

[9] Dweck CS (2008). Mindset: the new psychology of success.

[10] Bostock R, Kinnison T, May SA (2018). Mindset and its relationship to anxiety in clinical veterinary students. Vet Rec.

[11] Whittington RE, Rhind S, Loads D, Handel I (2017). Exploring the link between mindset and psychological well-being among veterinary students. J Vet Med Educ.

[12] Babenko O, Daniels LM, Ross S, White J, Oswald A (2019). Medical student well-being and lifelong learning: a motivational perspective. Educ Health.

[13] Calo M, Peiris C, Chipchase L, Blackstock F, Judd B (2019). Grit, resilience and mindset in health students. Clin Teach.

[14] Burnette JL, Knouse LE, Billingsley J, Earl S, Pollack JM, Hoyt CL (2023). A systematic review of growth mindset intervention implementation strategies. Soc Personal Psychol..

[15] Yeager DS, Hanselman P, Walton GM, Murray JS, Crosnoe R, Muller C (2019). A national experiment reveals where a growth mindset improves achievement. Nature..

[16] Williams C (2021). Nursing students’ mindsets and choice of learning strategies. Nurs Educ.

[17] Schleider J, Weisz J (2018). A single-session growth mindset intervention for adolescent anxiety and depression: 9-month outcomes of a randomized trial. J Child Psychol Psyc.

[18] OECD. PISA 2018 results (volume III): what school life means for students’ lives. 2019.

[19] Yeager DS, Dweck CS (2020). What can be learned from growth mindset controversies?. Am Psychol.

[20] Lewis LS, Williams CA, Dawson SD (2020). Growth mindset training and effective learning strategies in community college registered nursing students. Teach Learn Nurs.

[21] Makary MA, Daniel M (2016). Medical error—the third leading cause of death in the US. BMJ.

[22] Jegathesan M, Vitberg YM, Pusic MV (2016). A survey of mindset theories of intelligence and medical error self-reporting among pediatric housestaff and faculty. Bmc Med Educ..

[23] Cutumisu M, Brown MRG, Fray C, Schmolzer GM (2018). Growth mindset moderates the effect of the neonatal resuscitation program on performance in a computer-based game training simulation. Front Pediatr.

[24] Cook DA, Gas BL, Farley DR, Lineberry M, Naik ND, Cardenas Lara FJ (2019). Influencing mindsets and motivation in procedural skills learning: two randomized studies. J Surg Educ.

[25] Klein J, Delany C, Fischer MD, Smallwood D, Trumble S (2017). A growth mindset approach to preparing trainees for medical error. BMJ Qual Saf.

[26] Suldo SM, Shaunessy E, Hardesty R (2008). Relationships among stress, coping, and mental health in high-achieving high school students. Psychol Schools.

[27] Jia X, Hao L, He L, Li P, Liu M, Zhang Y, Qiu J (2023). Regional gray matter volume is associated with growth mindset: a voxel-based morphometry study. Neuroscience.

[28] Mangels JA, Butterfield B, Lamb J, Good C, Dweck CS (2006). Why do beliefs about intelligence influence learning success? A social cognitive neuroscience model. Soc Cogn Affect Neur.

[29] Geary RT (2014). The limbic system : anatomy, functions and disorders.

[30] Sun X, Nancekivell S, Gelman SA, Shah P (2021). Growth mindset and academic outcomes: a comparison of US and Chinese students. NPJ Sci Learn.

[31] Symon B. 127 The Ben Symon Special Edition: educational theories to change your life; 2021. Podcast.

[32] Muenks K, Canning EA, LaCosse J, Green DJ, Zirkel S, Garcia JA, Murphy MC (2020). Does my professor think my ability can change? Students’ perceptions of their STEM professors’ mindset beliefs predict their psychological vulnerability, engagement, and performance in class. J Exp Psychol Gen.

[33] Canning EA, Muenks K, Green DJ, Murphy MC (2019). STEM faculty who believe ability is fixed have larger racial achievement gaps and inspire less student motivation in their classes. Sci Adv.

